# Biodegradable nanoparticles sequentially decorated with Polyethyleneimine and Hyaluronan for the targeted delivery of docetaxel to airway cancer cells

**DOI:** 10.1186/s12951-015-0088-2

**Published:** 2015-04-03

**Authors:** Sara Maiolino, Annapina Russo, Valentina Pagliara, Claudia Conte, Francesca Ungaro, Giulia Russo, Fabiana Quaglia

**Affiliations:** Laboratory of Drug Delivery, Department of Pharmacy, University of Napoli Federico II, Via Domenico Montesano 49, Napoli, 80131 Italy; Laboratory of Biochemistry, Department of Pharmacy, University of Napoli Federico II, Via Domenico Montesano 49, Napoli, 80131 Italy

**Keywords:** Nanoparticles, CD44 targeting, Poly(lactic-co-glycolic) acid, Hyaluronan, Polyethyleneimine, Docetaxel, Lung cancer

## Abstract

**Background:**

Novel polymeric nanoparticles (NPs) specifically designed for delivering chemotherapeutics in the body and aimed at improving treatment activity and selectivity, cover a very relevant area in the field of nanomedicine.

Here, we describe how to build a polymer shell of Hyaluronan (HA) and Polyethyleneimine (PEI) on biodegradable NPs of poly(lactic-co-glycolic) acid (PLGA) through electrostatic interactions and to achieve NPs with unique features of sustained delivery of a docetaxel (DTX) drug cargo as well as improved intracellular uptake.

**Results:**

A stable PEI or HA/PEI shell could be obtained by careful selection of layering conditions. NPs with exquisite stability in salt and protein-rich media, with size and surface charge matching biological requirements for intravenous injection and endowed with sustained DTX release could be obtained. Cytotoxicity, uptake and activity of both PLGA/PEI/HA and PLGA/PEI NPs were evaluated in CD44(+) (A549) and CD44(−) (Calu-3) lung cancer cells. In fact, PEI-coated NPs can be formed after degradation/dissociation of the surface HA because of the excess hyaluronidases overexpressed in tumour interstitium. There was no statistically significant cytotoxic effect of PLGA/PEI/HA and PLGA/PEI NPs in both cell lines, thus suggesting that introduction of PEI in NP shell was not hampered by its intrinsic toxicity. Intracellular trafficking of NPs fluorescently labeled with Rhodamine (RHO) (RHO-PLGA/PEI/HA and RHO-PLGA/PEI NPs) demonstrated an increased time-dependent uptake only for RHO-PLGA/PEI/HA NPs in A549 cells as compared to Calu-3 cells. As expected, RHO-PLGA/PEI NP uptake in A549 cells was comparable to that observed in Calu-3 cells. RHO-PLGA/PEI/HA NPs internalized into A549 cells showed a preferential perinuclear localization. Cytotoxicity data in A549 cells suggested that DTX delivered through PLGA/PEI/HA NPs exerted a more potent antiproliferative activity than free DTX. Furthermore, DTX-PLGA/PEI NPs, as hypothetical result of hyaluronidase-mediated degradation in tumor interstitium, were still able to improve the cytotoxic activity of free DTX.

**Conclusions:**

Taken together, results lead us to hypothesize that biodegradable NPs coated with a PEI/HA shell represent a very promising system to treat CD44 overexpressing lung cancer. In principle, this novel nanocarrier can be extended to different single drugs and drug combinations taking advantage of the shell and core properties.

**Electronic supplementary material:**

The online version of this article (doi:10.1186/s12951-015-0088-2) contains supplementary material, which is available to authorized users.

## Background

In the past twenty years, nanotherapeutics have been introduced in the clinical practice for treating tumors with the goal to improve therapeutic outcome of conventional pharmacological therapies, to alleviate their toxicity as well as to overcome multidrug resistance [1-8]. By providing a protective housing for the drug, nanoscale delivery system can in theory offer the advantages of drug protection from degradation, efficient control of pharmacokinetics and accumulation in tumor tissue, thus limiting drug interaction with healthy cells and as a consequence side effects.

Polymer-based nanoparticles (NPs) specifically designed for cancer treatment cover a very relevant and widely explored area in the field of nanotechnology [9-11]. The main advantages of polymeric NPs reside in the opportunity to readily manipulate their properties by selecting polymer type and mode of carrier preparation. As a consequence, not only those surface features which affect biological behavior (spatial distribution of drug dose in the body) are controlled, but also timing of drug release (temporal control of drug availability to target) is predetermined.

Due to their well-established biocompatibility and safety profile, nano-oncologicals made of polyesters such as poly(lactic-co-glycolic) acid (PLGA) can be considered one of the most interesting systems for this application and are greatly emerging in the field [2]. To be used as an effective nanomedicine, PLGA NPs need to be surface-engineered according to specific technological and therapeutic needs [12]. Core-shell architecture represents an effective way to attain multiple functionalities on a nanoscopic length scale. Indeed, a core (template) generally carrying a chemotherapeutic agent can be surrounded by a shell with different composition and configuration that provides a functional and interacting interface with biological environment [13]. The shell is also responsible of colloidal stability of the system “in the bottle” (shelf life) and in biologically relevant media. A wide array of currently-available materials and possible combinations can be used to fabricate core-shell NPs spanning from tailored amphiphilic polymers, able to form nanoassemblies in aqueous media, to nanostructures where electrostatic or hydrophobic interactions drive shell deposition on a core template [9,11]. The latter approach is very attractive since no complicate synthetic steps to attain polymer functionalization are required.

In the construction of layered NPs, an emerging aspect in nanotechnology is represented by the use of polymers with specific functions for cancer treatment. Two relevant examples are Polyethyleneimine (PEI) and Hyaluronan (HA). PEI is a cationic polymer widely employed for transfection due to its capacity to complex polyanionic DNA and oligonucleotides (decoy, siRNA, miRNA)[14]. Recently, it has been demonstrated that PEI can induce anticancer effects via electrostatic interaction with cell membranes [15,16]. Furthermore, it has been observed that intratumoral injection of different cationic polymers evokes a robust infiltration of Th 1 and NK cells into the tumor site, reversing the tumor microenvironment from immunosuppressive to immunostimulatory and inducing massive tumor necrosis [17]. In other studies, PEI triggered Antigen Presenting Cell activation via TLR-signaling and exerted direct tumoricidal activity in a mouse model of ovarian tumors [18]. Cationic NPs are considered intriguing systems to promote deep penetration in tumor tissues [19,20]. Nevertheless, cationic NPs induce rapid formation of complex aggregates with negatively charged serum molecules or membranes of cellular components, which are then cleared by the reticuloendothelial system (RES). More importantly, many cationic nanosystems developed so far exhibit substantial toxicity [21], which has limited their clinical applicability thus demanding strategies to reversibly shield their surface with biomimetic coatings.

A functional anionic polymer such as hyaluronan (HA) can perfectly fit the purpose to cover cationic NPs through electrostatic interactions and to form a bioresponsive shell. HA is a negatively-charged polysaccharide with a relevant role in cancer since its receptors (CD44, RHAMN) are overexpressed on the surface of a broad variety of cancer cells [22]. Recently, combination of chemotherapeutic drugs with HA NPs for selective targeting of CD44-overexpressed cancer cells has received increasing attention to improve specificity of the drug and alleviate side effects [23]. In fact, receptor-mediated endocytosis of HA-decorated NPs facilitates drug transport inside the cells and contributes to enhanced drug cytotoxicity [24]. Nevertheless, HA is involved in extracellular matrix remodeling of cancer tissues since HA degradation by interstitium hyaluronidases (Hyals) is a process at basis of facilitated metastasis formation [23].

Although based only on electrostatic interactions, core-shell PLGA NPs prepared by stepwise modification through sequential layering of positive and negative polymers such as alginate/chitosan, alginate/polylysine and HA/polylysine [25], HA/polyarginine [26] as well as HA/PEI [27] has been found successful to achieve physically stable NPs for the co-delivery of different drug combinations.

In this paper, we describe how to build a polymer shell on biodegradable PLGA NPs through PEI or PEI/HA electrostatic interactions and achieve NPs with unique features of sustained delivery of their docetaxel (DTX) drug cargo and improved intracellular uptake. In fact, several DTX polymeric nanoplatforms have been developed so far to improve drug activity and selectivity as well as to overcome multi drug resistance [28-32]. After a formulation study aimed at optimizing preparation conditions of NPs, a powder for injection was obtained and characterized for release properties and stability in media with different ions and protein contents. PEI and HA/PEI-decorated NPs were tested for trafficking and activity in CD44(+) and CD44(−) lung cancer cells and potential contribute of HA targeting was highlighted.

## Results and discussion

To improve DTX activity and internalization in airway cancer cells, we tried to engineer biodegradable PLGA NPs with either a PEI coating able to confer a cationic charge to the system or a PEI/HA shell endowed with ability of CD44 receptor targeting. An anionic non end-capped PLGA nanoparticle template entrapping the model anticancer drug DTX was covered with a polycationic layer of PEI (25 kDa, branched). Further application of a finishing layer of low molecular weight HA (<10 kDa) was accomplished to allow CD44 receptor targeting. We first developed a robust layering procedure to obtain NPs and then characterized the system in biologically-relevant media. Next, cytotoxicity and uptake of the unloaded NPs, as well as activity of DTX-loaded NPs in CD44(+) and CD44(−) lung cancer cells were assessed also considering a possible HA shedding and generation of PEI-coated NPs.

### Layering procedure

**Figure 1:**
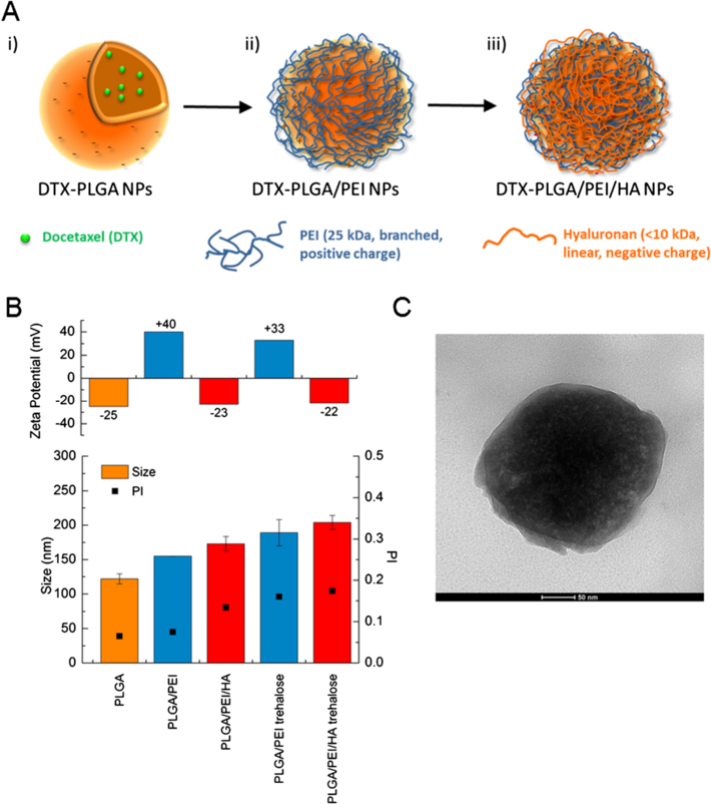
**Structure and properties of NPs during layering procedure. A)** Schematic representation of the double coating process. **B)** Size, polydispersity (PI) and zeta potential of NPs during coating and after dispersion of NPs freeze-dried with trehalose in water. Results are the mean of three measurements obtained on three different NP batches ± SD. **C)** TEM micrographs of PLGA/PEI/HA NPs.

Preparation of DTX-loaded PLGA/PEI/HA NPs is a multistep process comprising i) the preparation of a PLGA core template; ii) the absorption of a positive PEI layer; iii) the absorption of a negative bioresponsive layer of HA (Figure 1A). DTX-PLGA NPs were prepared by nanoprecipitation of a PLGA acetone solution in a Pluronic F68 water solution. The size of unloaded PLGA NPs was only slightly affected by PLGA concentration while the surface was always negative (Additional file 1: Figure S1A). Thus, a PLGA concentration of 5 mg/mL was selected for all further experiments. After a washing step to eliminate surface-adsorbed Pluronic F68, around 60% of NPs with the original size/zeta potential were recovered. Then, a first positive layer of PEI was adsorbed through electrostatic interactions by incubating NPs with a PEI water solution.

The process of polymer film growth onto NP surface can be severely altered by several experimental factors and mainly by salt concentration in the medium [33]. PEI layering was thereby carried out both in water and PBS 0.01 M at pH 7.4. However, only water allowed the prompt re-dispersion of PLGA/PEI NP pellet without aggregation. Thus, all the preparation steps were carried out in water. The addition of 125 μL of a PEI water solution at increasing concentration (0.5, 1.0 and 1.5 mg/mL) caused an inversion of zeta potential from negative to positive values (Additional file 1: Figure S1B). Optimal conditions for layering were found at 1 mg/mL since only a slight increase of size and polydispersity index (PI) was observed (Figure 1B). PLGA/PEI NPs were further collected to eliminate un-adsorbed PEI and dispersed in water again. In the following step, PLGA/PEI NPs were finished with a HA layer giving PLGA/PEI/HA NPs, which displayed a slightly increased size and negative zeta potential. Quantitative adsorption of HA on NP surface was confirmed by evaluating the amount of HA in the solution after collecting NPs (no free HA was detected in the medium).

In developing translational NPs for intravenous route, it is of key importance to produce a lyophilized NP powder with a suitable shelf-life and with effective dispersion in the diluting vehicles usually employed for administration. While PLGA/PEI and PLGA/PEI/HA NPs freeze-dried as such underwent extensive aggregation and could not be dispersed in water (data not shown) the addition of a cryoprotectant such as trehalose gave a powder that, after dispersion in water, restored the original NP size, PI and zeta potential (Figure 1B).

### Properties of DTX-loaded NPs

Overall properties of DTX-loaded PLGA/PEI and PLGA/PEI/HA NPs are reported in Table 1. DTX entrapment in the PLGA core did not alter size, PI and zeta potential also after the freeze-drying step. Around 50% of the initial PEI amount was retained on NP surface whereas the absorption of HA was complete. The yield of the production process was found to be around 20% (Table 1), which is in line with values reported for layered NPs. It is worth of note that final NPs can be loaded with amount of DTX in the PLGA core up to 4 mg/100 mg NPs without altering size and zeta potential (Additional file 1: Table S1). These results are in line with those found for DTX entrapped in PLGA NPs prepared by nanoprecipitation [34]. TEM analysis confirmed the spherical morphology and lack of aggregation of the final formulation (Figure 1C).

**Table 1:** **Properties of DTX-loaded NPs before and after freeze-drying**

	**DTX-PLGA/PEI NPs**	**DTX-PLGA/PEI/HA NPs**	**DTX-PLGA/PEI NPs freeze-dried**	**DTX-PLGA/PEI/HA NPs freeze-dried**
Trehalose/NP weight ratio	-		60	
Yield (% ± SD)	21.2 ± 0.8^a^		-	
Mean D_H_ (nm ± SD)	155 ± 9^b^	173 ± 10^b^	176 ± 4^c^	203 ± 10^c^
Polydispersity index	0.10^b^	0.13^b^	0.20^c^	0.17^c^
Zeta Potential (mV)	+39.0^b^	−23.2^b^	+33.5^c^	−22.3^c^
DTX actual loading(mg *per* 100 mg)	0.95 ± 0.5		0.013 ± 0.001	
PEI actual loading(mg *per* 100 mg)	7.94 ± 2.1		0.132 ± 0.010	
HA actual loading(mg *per* 100 mg)	-	12.2 ± 0.8	-	0.20 ± 0.03

^a^Calculated as ratio between total component weight and NP weight.

^b^As prepared NPs before freeze-drying.

^c^After dispersion of freeze-dried powder in water.

**Figure 2:**
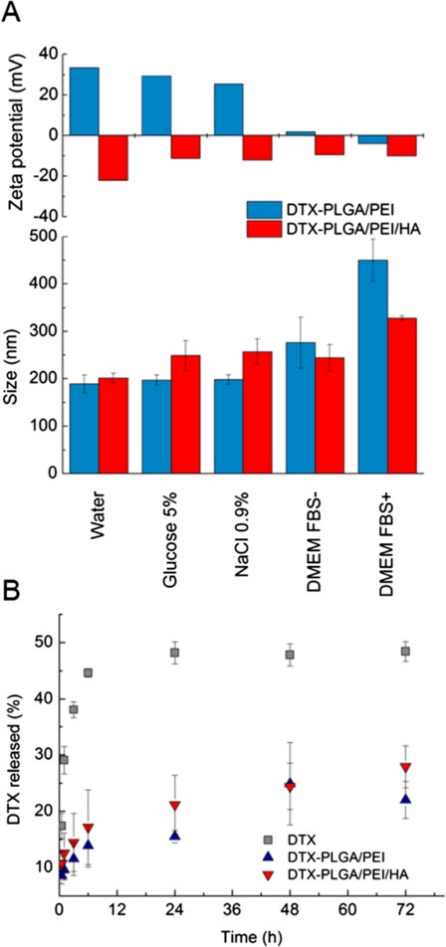
**Properties of DTX-PLGA/PEI and DTX-PLGA/PEI/HA NPs freeze-dried with trehalose. A)** Size and zeta potential in different media. **B)** Release profile (37°C) of DTX from NPs dispersed in DMEM FBS+. External dialysis medium was a PBS solution at pH 7.4. Free DTX is reported as control. Results are the mean of three experiments ± SD.

A drawback with a layering procedure carried out in water could be the occurrence of weaker interactions between negative and positive polymer chains and, as a consequence, the formation of a loose and poorly stable coating. On these bases, we tested the stability of DTX-PLGA/PEI and DTX-PLGA/PEI/HA NPs in different media by measuring size and zeta potential (Figure 2A). Both types of NPs were stable in NaCl 0.9% or glucose 5% solution giving size and zeta potential values in line with those found in water. To mimic more closely NP behavior in cell culture experiments, stability was also assessed in DMEM without or with FBS (FBS- and FBS+, respectively). The results suggested that DTX-PLGA/PEI NPs greatly increase their size presumably due to extensive protein adsorption while size increase of DTX-PLGA/PEI/HA NPs was limited. Size growth in the presence of proteins did not give macroscopic NP aggregation over time. Zeta potential of DTX-PLGA/PEI NPs in cell culture media switched to values very close to neutrality independently of the presence of proteins.

Release profile of DTX from both NPs type was evaluated by dialysis using DMEM FBS+ as dispersing medium and PBS at pH 7.4 as external medium (sink conditions were set). DTX release from DTX-PLGA/PEI and DTX-PLGA/PEI/HA NPs was monitored for 72 h and compared with that of free DTX (Figure 2B). The transport of free DTX toward the external medium was found to be incomplete in this experimental set-up due to strong DTX-protein interaction inside the dyalisis bag hampering DTX transport in the external medium. This behavior is in agreement with previous data collected on DTX solubilized in human plasma [35]. However, a slow and sustained release of DTX from both DTX-PLGA/PEI and DTX-PLGA/PEI/HA NPs as compared with free DTX was found. The release rate observed for coated NPs was similar thus suggesting that for an unionized drug such as DTX, the presence of the polyelectrolyte layer does not represent a further barrier to drug transport from the PLGA core.

### Cytotoxicity of unloaded NPs

The cytotoxicity was evaluated on PLGA/PEI/HA and PLGA/PEI NPs, taking into account that NP shedding due to HA degradation can occur in a biological environments. Indeed, several cancers produce elevated levels of Hyals. A higher expression level of Hyals was also found in metastatic tumors as compared to non-metastatic [23]. In principle, cationic NPs can be formed by dissociation of the surface HA because of the excess Hyals in tumor interstitium.

The cytotoxicity was assessed by the 3-(4,5-dimethylthiazol-2-yl)-2,5-diphenyltetrazolium bromide (MTT) and lactate dehydrogenase (LDH) release assays. The experiments were performed in A549 and Calu-3 cells selected as CD44(+) and CD44(−) cell lines, respectively [36]. Cells were incubated with a wide range of NP concentrations (from 0.1 mg to 2 mg) and tested upon 24 h and 72 h of treatment.

**Figure 3:**
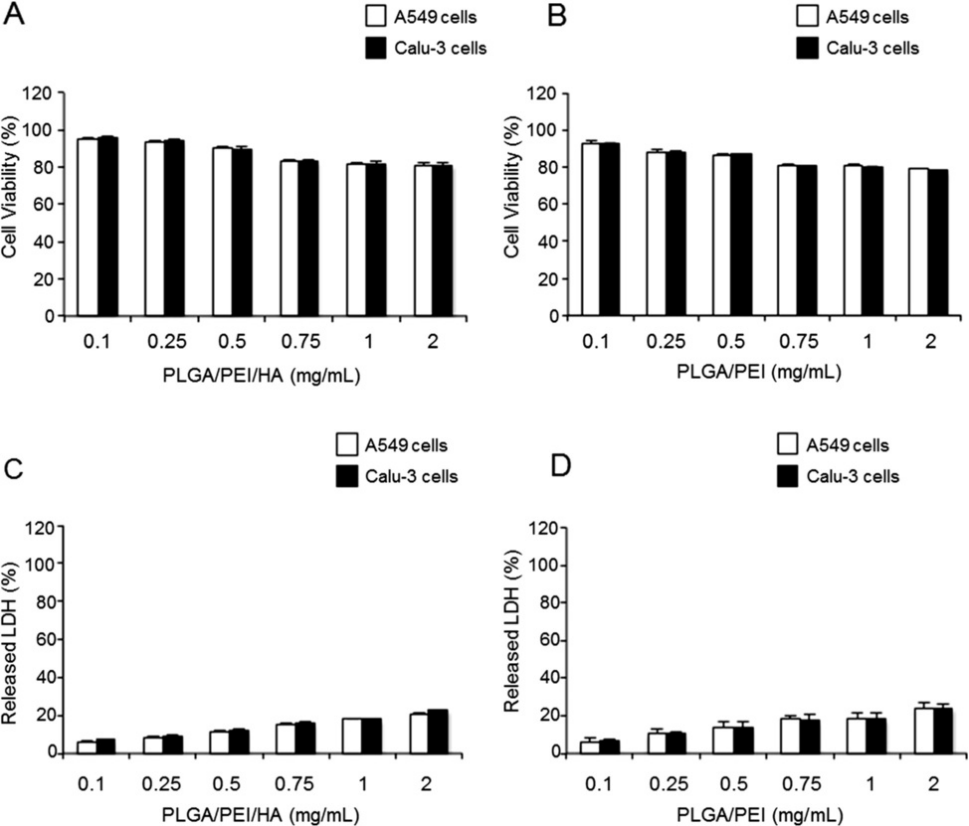
**Cytotoxicity of unloaded NPs.** A549 and Calu-3 cells were exposed to increasing concentrations of PLGA/PEI/HA and PLGA/PEI NPs for 24 h. After incubation, cell viability and released LDH were evaluated using the MTT **(A, B)** and LDH **(C, D)** assays. The cell viability and LDH release from untreated control were set to 100% and 0%, respectively. Results are presented as percentage (mean ± SEM) (*n* = 3) of the control cells. Differences were considered not statistically significant (*P* >0.05).

Results from MTT assay in Figure 3A show that 24 h after the treatment, A549 and Calu-3 cells retained about 90% viability when treated with various concentrations of PLGA/PEI/HA NPs as compared with control (untreated cells set to 100%). At 24 h, the cytotoxicity curve for PLGA/PEI NPs followed an almost similar pattern (Figure 3B). The observed results were confirmed by the values of cell mortality from LDH assay. A549 and Calu-3 cells were incubated with the same concentrations of PLGA/PEI/HA or PLGA/PEI NPs and cell membrane damage was assessed 24 h later (Figure 3C and D). As shown, the results obtained from the LDH assay were in line with those obtained with the MTT assay.

Data obtained from MTT and LDH assays after 72 h of treatment with PLGA/PEI/HA and PLGA/PEI NPs demonstrated no significant NP toxicity (Additional file 1: Figure S2) analogously to PLGA NPs (Additional file 1: Figure S3).

All these results clearly showed that there was no statistically significant cytotoxic effect (P >0.05) of both PLGA/PEI/HA and PLGA/PEI NPs in either A549 or Calu-3 cell lines and supported the hypothesis that PEI strongly decreases its cytotoxicity when delivered through NPs presumably due to a depression of cationic charge in the medium [37,38].

### Uptake of NPs

**Figure 4:**
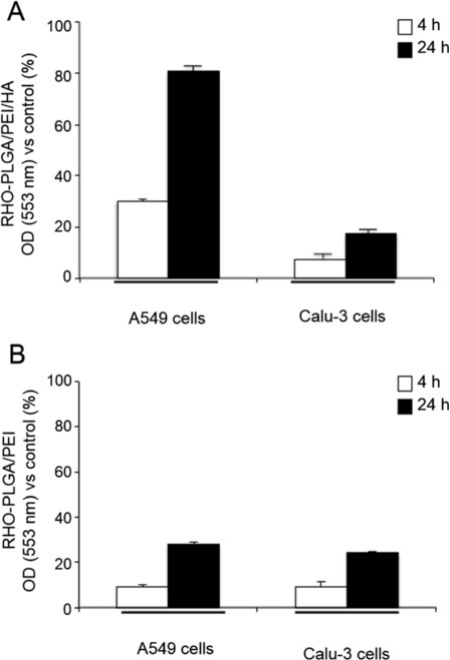
**Cellular uptake of fluorescent NPs.** Intracellular levels of fluorescent RHO-PLGA/PEI and RHO-PLGA/PEI/HA NPs. A549 and Calu-3 cells were incubated with 0.5 mg/ml of RHO-PLGA/PEI/HA NPs **(A)** and RHO-PLGA/PEI NPs **(B)** for 4 h and 24 h. All measurements were normalized to the fluorescence of RHO-labeled NPs in cell medium set as 100%. Results are presented as percentage (mean ± SEM) (*n* = 3) of the control cells.

The natural turnover of free HA is predominantly based on its CD44 receptor-mediated internalization in cells [39]. Starting from this knowledge, we became interested to study the intracellular uptake of PLGA/PEI/HA and PLGA/PEI NPs in A549 (CD44(+)) and Calu-3 (CD44(−)) cells. To this aim, fluorescent NPs labeled with Rhodamine (RHO), named RHO-PLGA/PEI/HA and RHO-PLGA/PEI NPs, were prepared from a RHO-PLGA covalent derivative (see Additional file 1: Figure S4). Properties of fluorescent RHO-NPs were comparable to those of untagged NPs (see Additional file 1: Table S2). Next, A549 and Calu-3 cells were incubated with RHO-PLGA/PEI/HA and RHO-PLGA/PEI for 4 h and 24 h. The fluorescence intensity of the internalized RHO-NPs was measured by fluorimetry. As shown in Figure 4 A, in A549 cells the treatment with RHO-PLGA/PEI/HA NPs was associated to an increasing time-dependent cellular uptake (30% and 80% after 4 h and 24 h, respectively). The observation that the lack of CD44 receptor did not provoke any significant uptake of RHO-PLGA/PEI/HA NPs in Calu-3 cells confirmed that the uptake of HA-decorated NPs was CD44 receptor-dependent. These results suggest that the endocytosis mechanism of PLGA/PEI/HA NPs internalization depends on the presence of HA onto the surface. As expected, RHO-PLGA/PEI NP uptake in A549 cells was comparable to that observed in Calu-3 cells (Figure 4B). However, it is important to note that RHO-PLGA/PEI NPs were still able to penetrate into cancer cells, giving about 10% and 35% NP uptake after 4 h and 24 h of treatment, respectively (Figure 4B). This finding is very intriguing in the light of recent data demonstrating that in tumor microenvironment HA-coated NPs could be converted to uncoated NPs as results of Hyals-mediated degradation [20]. In perspective, loss of HA coating can allow in situ generation of cationic NPs, which are still able to effectively enter inside cancer cells.

**Figure 5:**
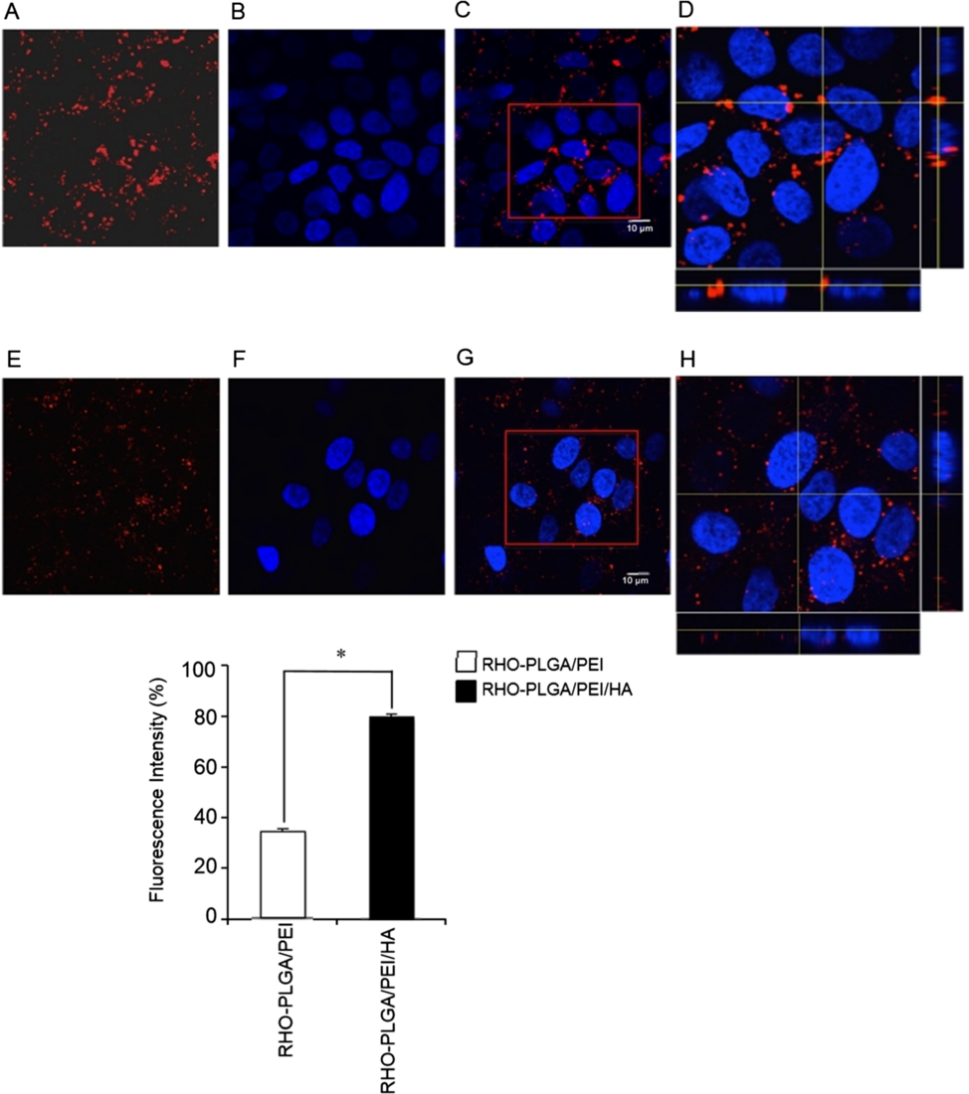
**Confocal microscopy images of A549 cells after incubation with fluorescent NPs.** A549 cells were incubated with RHO-PLGA/PEI/HA NPs **(A)** and RHO-PLGA/PEI NPs **(E)** for 24 h. Confocal microscopy images 100X: A549 cell nuclei stained with DAPI **(B, F)**. Merge of the same field for composite images **(C, G)**, scale bar = 10 μm. Pictures were processed using ImageJ Software to reconstruct the x-axis projection using stack images **(D, H)**. Quantification of fluorescence intensity is shown. Bars represent mean values ± SEM of experiments done in triplicate. *P < 0.05.

Next, to investigate the subcellular distribution of internalized NPs, we carried out confocal laser scanner microscopy (CLSM) analysis into A549 cells (Figure 5 and Additional file 1: Figure S5). After incubation with RHO-PLGA/PEI/HA NPs (Figure 5A) and RHO-PLGA/PEI NPs (Figure 5E), the cells were stained with DAPI (Figure 5B and F) and observed under the microscope. A large majority of the RHO-PLGA/PEI/HA NPs-associated fluorescence appeared to be distributed in the vicinity, and surrounding, of the cells nuclei, confirming the internalization of NPs (Figure 5C). The x-axis projections are shown in Figure 5D. Analysis of z-sections taken through the cell nucleus showed the absence of any fluorescent signal for both types of NPs (see Additional file 1: Figure S6). It may be noticed that the perinuclear accumulation of NPs after internalization in cells can provide a sustained drug delivery in the proximity of the nucleus. This feature might be interesting for the development of tumor suppressor gene-loaded NPs as apoptosis-induction adjuvant, aimed at improving the outcome of common anticancer therapies as 5-FU and L-OHP [40]. Internalized RHO-PLGA/PEI NPs (Figure 5G and H) were indeed more homogeneously distributed in the whole cytoplasm. The quantification of intracellular fluorescence demonstrated the efficacy of HA to mediate RHO-PLGA/PEI/HA NP internalization (about 80%) as compared to non-targeted RHO-PLGA/PEI NPs (about 30%).

**Figure 6:**
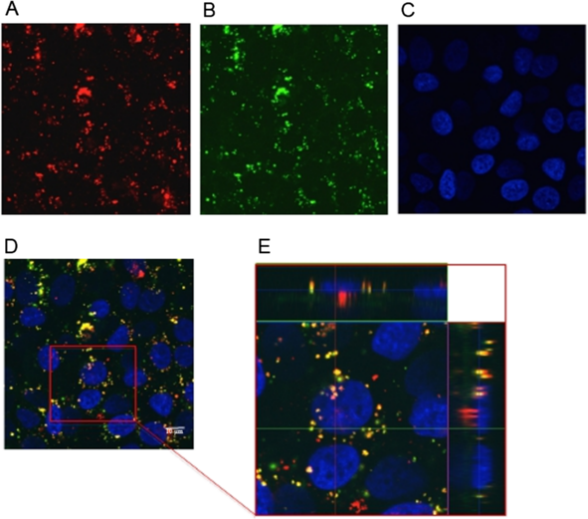
**Subcellular distribution of fluorescent NPs in A549 cells.** A549 cells were incubated with RHO-PLGA/PEI/HA NPs for 24 h. Confocal microscopy images 100X: RHO-PLGA/PEI/HA NPs **(A)**, lysosomes of A549 cells stained with LysoTracker Green **(B)**, A549 cell nuclei stained with DAPI **(C)**. Merge of the same field for composite images **(D)**, scale bar = 10 μm. Pictures were processed using ImageJ Software to reconstruct the x-axis projection using stack images **(E)**. Composite image demonstrated colocalization of RHO-PLGA/PEI/HA NPs with lysosomes.

In addition to internalization mechanisms, the intracellular trafficking of NPs within the cell are critical elements to study when a new drug carrier is proposed. Among these issues, the subcellular distribution of the NPs represents an important aspect of NP dynamics. Lysosomes are a common terminal degradative compartment of certain endocytotic pathways. Thus, understanding whether NPs are delivered to the lysosomes following their internalization is a key point [28]. To determine whether the internalized RHO-PLGA/PEI/HA NPs were addressed to the lysosomal compartments, A549 cells were incubated with fluorescent RHO-PLGA/PEI/HA NPs for 24 h (Figure 6A) and then stained with LysoTracker Green (Figure 6B) and DAPI (Figure 6C). As shown in Figure 6D, a partial colocalization of RHO-PLGA/PEI/HA NPs with lysosome, as evident from the appearance of orange to yellow fluorescence, was observed. The x-axis projection was also shown (Figure 6E). This finding suggests that both different entry mechanisms of NPs and a possible NP escape from lysosomes can contribute to their intracellular distribution, which needs to be addressed in further studies.

### Cytotoxicity of DTX-loaded NPs

**Figure 7:**
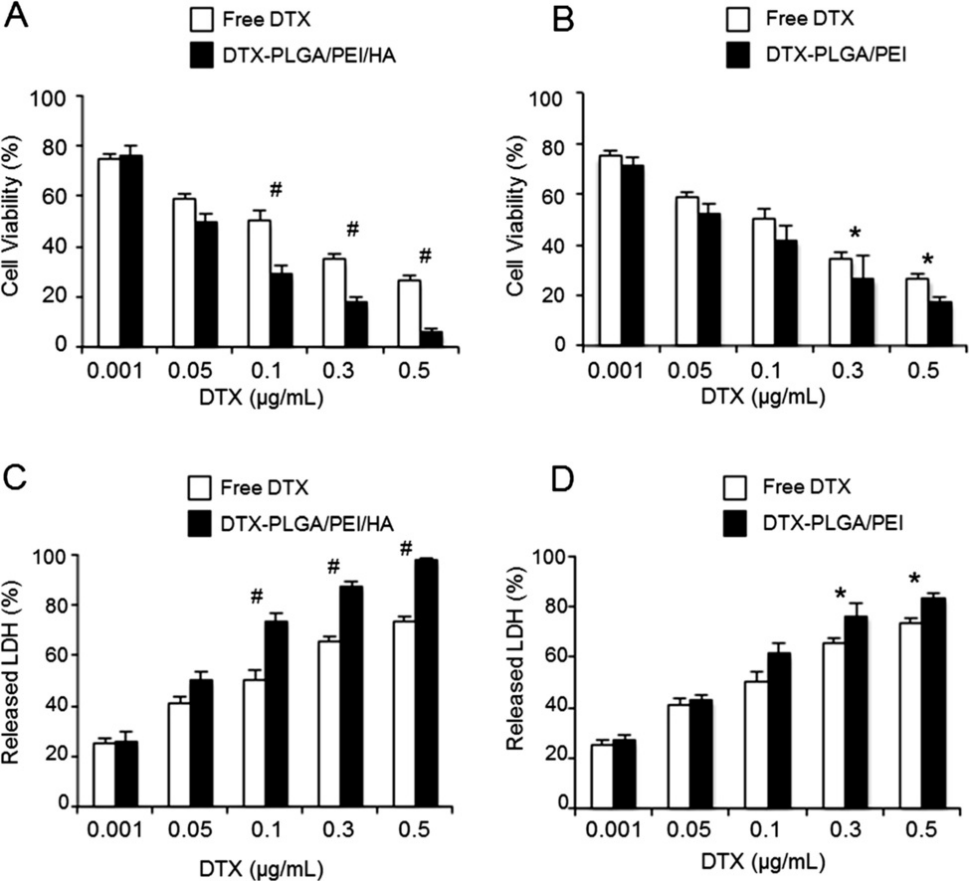
**Cytotoxicity of DTX loaded-NPs in A549 cells.** A549 cells were exposed to increasing concentrations of free DTX, DTX-PLGA/PEI/HA NPs or DTX-PLGA/PEI NPs for 72 h. After incubation, cell viability and released LDH were evaluated using the MTT **(A, B)** and LDH **(C, D)** assays. The cell viability and LDH release from untreated cells were set to 100% and 0%, respectively. Results are presented as percentage (mean ± SEM) (*n* = 3) of the control cells. *P <0.05, ^#^ *P* < 0.001.

Once the biological behavior of NPs was characterized, we tested their ability to improve the activity of DTX. *In vitro* cytotoxicity of DTX-PLGA/PEI/HA and DTX-PLGA/PEI NPs was evaluated in A549 cells after 24 h and 72 h exposure by using MTT and LDH assays and compared to that of free DTX. Extrapolation of the dose causing 50% cell death (IC_50_) from the dose–response curve at 72 h (Table 2) showed that in A549 cells treated with DTX-PLGA/PEI/HA NPs activity was two–fold higher than that of free DTX. Nevertheless, an activity similar to that of free DTX was found in Calu3 cells. DTX-PLGA/PEI NPs displayed an activity comparable to that of free DTX in both cell lines, although fractional drug release occurred. The observed results were confirmed by the values from LDH assay (Figure 7C). Results from MTT and LDH assays of DTX-PLGA/PEI NPs at 72 h (Figure 7B and D) showed that the cytotoxicity induced by DTX-PLGA/PEI NPs was increased of about 15% as compared with free DTX. Data from MTT and LDH assays at 24 h (Additional file 1: Figure S7) show a similar trend.

**Table 2:** **IC**_**50**_ **values of free DTX, DTX-PLGA/PEI and DTX-PLGA/PEI/HA NPs on A549 cells following 24 and 72 h incubation (n = 3)**

	**IC**_**50**_ **(μg/mL)**		
	DTX	DTX-PLGA/PEI NPs	DTX-PLGA/PEI/HA NPs
24 h	0.5 ± 0.048	0.48 ± 0.089	0.3 ± 0.030
72 h	0.1 ± 0.062	0.07 ± 0.072	0.05 ± 0.040

The increase of DTX activity when delivered from NPs can be explained on the basis of its influx/efflux from cells as well as its mode of action. Concerning influx/efflux, although the exact mechanism of DTX entry inside cells (passive diffusion, active transporters, free DTX, protein-bound) is not clear, it has been demonstrated that effective uptake of NPs can well correlate with DTX activity. After NP entry, DTX is released, binds tubulin and stabilizes microtubules, leading to cell cycle arrest at G2M phase and further to initiation of apoptosis and cytotoxicity [41], It is possible that slow intracellular release of DTX from NPs is much more controlled than a single extracellular dose where above a certain DTX level, a saturation of binding site for tubulin occurs and drug efflux start taking place [42]. Furthermore, efflux of DTX can be hampered by NPs, which is in line with reversion of multi Drug resistance observed in vitro and in vivo when employing DTX nanoformulations [43].

Taken together, results lead us to hypothesize that once the DTX-PLGA/PEI/HA NPs reach the CD44-overexpressed tumor site, they could be uptaken through CD44 receptor, be distributed to different critical compartments where more efficient drug release as compared with the free DTX form is achieved. Nevertheless, DTX-PLGA/PEI NPs, as hypothetical result of Hyals-mediated degradation in tumor [20] are still able to improve the cytotoxic activity of free DTX contributing in this way to further increase the therapeutical potential of original DTX-PLGA/PEI/HA NPs.

## Conclusion

This study has demonstrated that it is possible to build CD44-targeted NPs with sustained drug delivery by electrostatic assembly of proper polymer building blocks. HA-coated NPs with excellent stability in complex media and demonstrating unprecedented uptake and activity in CD44-overexpressing lung cancer cells as compared with free drug were obtained. Cationic NPs as such, or generated after possible HA shedding in tumor interstium, displayed a higher cytotoxicity than that of free drug, further highlighting the therapeutic potential of the whole nanocarrier. Taken together, results lead us to hypothesize that biodegradable NPs developed here represent a very promising system for the targeted delivery of different single drugs and drug combinations taking advantage of the shell and core properties.

## Methods

## Materials

Docetaxel (DTX, MW = 807.88) was purchased from LC laboratories (USA). Hyaluronan (HA, <10 kDa) was a kind gift of Magaldi Life S.r.l. (Italy). Poly(lactic-*co*-glycolic) acid (PLGA) (D,L-lactic, 50:50 Resomer RG 502H, inherent viscosity 0.16 - 0.24 dl/g) was purchased from Boehringer Ingelheim (Germany). Rhodamine B (RHO), trehalose, polyethyleneimine (PEI, 25 kDa branched), copper (II) sulfate, hexadecyltrimethyl-ammonium bromide, poloxamer 188 (Pluronic® F68), sodium acetate, sodium chloride, sodium hydroxide, glacial acetic acid and trifluoracetic acid (TFA) were purchased from Sigma-Aldrich. Acetonitrile and acetone were purchased from Carlo Erba Reagenti (Italy). DMEM and Fetal Bovine Serum (FBS) were purchased from GibcoLife Technologies. Distilled water was used throughout the study.

### Preparation of DTX-PLGA/PEI/HA NPs

NPs were prepared through a layer-by-layer deposition method. In a first step, DTX-PLGA NPs were prepared by nanoprecipitation. PLGA 502H (5 mg) and DTX (5% w/w ) were co-dissolved in acetone (1 mL) and added drop-wise to 2 mL of an aqueous phase containing Pluronic F68 (0.1% w/v) under magnetic stirring. The organic solvent was then evaporated under vacuum using a rotary evaporator for 15 min. Thereafter, NP dispersion was splitted in 4 Eppendorf® tubes and centrifuged at 5000 x g for 15 min (Mikro 20 Zentrifugen). In the second step, NP pellet in each Eppendorf® tube was disperded in 1 mL of ultrapure water (final PLGA NP concentration was 1.5 mg/mL) and in each sample 125 μL of a PEI solution in water (0.5, 1.0 and 1.5 mg/mL) were added under stirring for 15 min. Thereafter, each sample was centrifuged again (2.800 x g for 10 min) to eliminate unadsorbed PEI and redispersed in 1 mL of ultrapure water (DTX-PLGA/PEI). In the third step, 100 μL of a HA solution (1 mg/mL) were added to PLGA/PEI NPs under stirring for 15 min (DTX-PLGA/PEI/HA). Final NPs were freeze-dried for 24 h with trehalose as cryoprotectant (30 mg in each vial) and kept at 4°C. Recovery yield of production process was evaluated on an aliquot of DTX-PLGA/PEI/HA NPs (without cryoprotectant) by weighting the solid residue after freeze-drying. Results are expressed as the ratio of the actual NPs weight to the theoretical polymer weight × 100.

Fluorescent NPs were prepared analogously by incorporating a 20% w/w of PLGA-RHO (see supplementary material S2) to PLGA when forming the core.

### Characterization of DTX-loaded NPs

#### Size, surface charge and morphology

Hydrodynamic diameter, polydispersity index (PI) and Zeta potential of NPs after each preparation step were determined on a Zetasizer Nano Z (Malvern Instruments Ltd., UK). Results are reported as mean of three separate measurements on three different batches  ± SD.

Particle morphology was analyzed on a NP dispersion in water (1 mg/mL) by Transmission Electron Microscopy (TEM) (CM 12 Philips, The Netherlands)

#### DTX entrapment efficiency

DTX loading inside DTX-PLGA/PEI/HA NPs was assessed by placing 0.5 mg of freeze-dried NPs or 30.5 mg of NPs freeze-dried with trehalose in 500 μL of DCM under stirring. The sample was dried and then 500 μL of water and 500 μL of acetonitrile were added under stirring. The sample was finally filtered through a 0.45 μm filter (RC, Chemtek, Italy).

DTX was analyzed by HPLC on an apparatus equipped with a LC-10ADvp pump, a SIL-10ADvp autoinjector, a SPD-10Avp UV–Vis detector and a C-R6 integrator (Shimadzu, Japan). The analysis was performed on a Supelco 5 μm, C18 column (250 × 4.6 mm, Å). The mobile phase was a 55:45 (v/v) mixture of water with TFA 0.1% and acetonitrile pumped at a flow rate of 1 mL/min. The UV detector was set at 227 nm. A calibration curve for DTX in ethanol was constructed in the concentration range 0.980–196 μg/mL. The limits of quantification (LOQ) and detection (LOD) were 1.29 and 0.39 μg/mL, respectively.

#### PEI amount in NPs

PEI was quantified by a colorimetric method developed previously [44]. To evaluate the amount of PEI in DTX-PLGA/PEI/HA NPs, 0.5 mg of freeze-dried NPs (with and without 30 mg of cryoprotectant) were treated with 1 mL of NaOH 1 M and stirred overnight. The sample (0.5 mL) was diluted with 0.5 mL of 1 M acetic acid. The resulting solution (0.5 mL) was added to 1 mL of acetate buffer 0.1 M at pH 5.4, and complexed with 0.25 mL of a copper (II) sulfate water solution (0.1% w/v). The absorbance value of each solution was recorded at 281 nm (UV 1800, Shimadzu, Japan). A calibration curve was constructed in the same condition in the PEI concentration range 15–380 μg/mL.

#### HA amount in NPs

To evaluate the extent of HA adsorption onto DTX-PLGA/PEI/HA NPs, 0.5 mg of NPs were centrifuged at 13000 x g for 15 min, the supernatant was withdrawn and freeze-dried. The solid residue was then dissolved in 1 mL of 0.2 M acetate buffer (0.2 M sodium acetate and 0.15 M sodium chloride) at pH 6. Thereafter, 2 mL of cetyltrimethylammonium bromide reagent (2 g sodium hydroxide, 1 g hexadecyltrimethylammonium bromide in 100 mL water) were added and the sample was analyzed at 350 nm. A calibration curve was constructed in the HA concentration range 10–200 μg/mL.

#### Stability in different media

Freeze-dried DTX-PLGA/PEI and DTX-PLGA/PEI/HA NP powder (30.5 mg corresponding to 0.5 mg of NPs) were dispersed in glucose 5%, NaCl 0.9%, DMEM without and with FBS 10% (1 mL). Size, polydispersity index and zeta potential of the samples were evaluated.

#### In vitro release studies

In vitro release of DTX from NPs was assessed in 10 mM phosphate buffer containing NaCl (137 mM) and KCl (2.7 mM) at pH 7.4 (PBS) by dialysis. Around 100 mg of DTX-PLGA/PEI and DTX-PLGA/PEI/HA freeze-dried powder (corresponding to 2 mg of NPs) were dispersed in 0.5 mL of DMEM with 10% FBS and placed in a dialysis bag (MWCO = 3500 Da, Spectra/Por®). The sample was plunged in PBS and kept at 37°C up to 72 h. At selected time intervals, 1 mL of release medium was withdrawn and replaced with an equal volume of fresh medium. DTX quantitative analysis was performed as described above. Release profile of free DTX dissolved in EtOH (10 μL, 2 μg/μL) and added to 0.5 mL of medium was assessed for comparison. Results are expressed as % release over time ± SD of three experiments.

### Cell cultures and treatments

A549 and Calu-3 cell lines were cultured in Dulbecco’s Modified Eagle’s Medium (DMEM) supplemented with 10% heat-inactivated fetal bovine serum (FBS) (Invitrogen, Life Technologies, Italy), 1.5 mM L-glutamine, 100 units/mL penicillin, and 100 μg/mL streptomycin under humidified atmosphere of 5% CO_2_ at 37°C. Treatments of cells were performed replacing the culture medium with those containing increasing concentrations of DTX (0.001 - 0.5 μg/mL), unloaded and DTX-loaded PLGA/PEI and PLGA/PEI/HA NPs (0.1 – 2 mg/mL of NPs). DMSO 0.1% (v/v) was used as vehicle for DTX.

### MTT assay

A549 and Calu-3 cells were seeded onto 96-well plates (2 × 10^4^ cells/well) and incubated with NPs for 24 h and 72 h. Then, cell viability was evaluated as mitochondrial activity using the MTT [45]. The absorbance was measured at 540 nm using a microplate reader (Labsystems Multiskan).

### LDH assay

LDH leakage into the media, an indicator of cell injury, was detected using the cytotoxicity assay, CytoTox 96® (Promega, USA), according to the manufacturer’s instructions, as described [46]. Samples from clarified medium of treated and untreated A549 and Calu-3 cells were taken after 24 h and 72 h of incubation and the LDH activity was measured.

### Confocal microscopy

A549 cells and Calu-3 cells were plated on coverslips at a density of 2 × 10^4^ cells per well in 12-well plates as previously reported [46]. Images of fluorescence-labeled cells were captured with a Zeiss LSM 510 meta confocal microscope equipped with an oil immersion plan Apochromat 100× objective 1.4 NA.

The laser line was set at 553 nm for RHO and 405 nm for DAPI. Images were acquired simultaneously in red and blue channels, and as z-stack. A gallery of optical slices was collected and *xz*, *yz* composites were processed using ImageJ Software to reconstruct the x-axis projection using stack images. The scale bars on all the images correspond to 10 μm.

The uptake of NPs was evaluated by measuring the incorporation of the fluorescent probe RHO in A549 and Calu-3 cells. Briefly, cells (2 × 10^4^ cells per well in 12-well plates) were collected and analysed by fluorimetry. The fluorescence was measured with a Cary Eclipse fluorescence spectrophotometer (Varian). Excitation and emission wavelengths were 553 and 627 nm, respectively.

### Statistical analysis

Error bars represent mean ± SEM from n = 3 biological replicates. Statistical comparisons were made by one-way ANOVA followed by Bonferroni’s test for multiple comparisons. *P <0.05 was considered significant, ^#^*P* <0.001 was considered highly significant. *P* >0.05 was considered not statistically significant.

## Additional file

Additional file 1: **Supplementary material.**
