# Supplementary material for: Biodegradable nanoparticles sequentially decorated with Polyethyleneimine and Hyaluronan for the targeted delivery of docetaxel to airway cancer cells
Source: J Nanobiotechnology. 2015 Apr 3;13:29. doi: 10.1186/s12951-015-0088-2 (PMC4424546; doi:10.1186/s12951-015-0088-2)
Supplement: Supplementary file 1 — Supplementary material. [file 12951_2015_88_MOESM1_ESM.docx]

**Supplementary material**

**Table S1.** Entrapment efficiency of DTX at different loading into PLGA NPs.

| **DTX theoretical loading**  **(% w/w)** | **Hydrodynamic diameter**  **(nm ± SD)** | **Zeta potential**  **(mV)** | **Actual loading**  **(mg/100 mg)^a^** | **Entrapment efficiency (% w/w)^b^** |
| --- | --- | --- | --- | --- |
| 5 | 123 ± 10 | -22 | 1.3 ± 0.5 | 26.5 |
| 10 | 124 ± 3 | -21 | 2.7 ± 1.0 | 27.2 |
| 15 | 128 ± 3 | -38 | 4 ± 2.4 | 26.6 |

^a^ Actual loading is expressed as the amount (mg) of drug encapsulated per 100 mg of NPs

^b^ Ratio between actual and theoretical loading×100.

**Table S2.** Properties of fluorescent NPs**.**

| **NP formulation** | **Hydrodynamic diameter**  **(nm±SD)** | **PI** | **Zeta potential (mV)** |
| --- | --- | --- | --- |
| RHO-PLGA | 138 ± 0.11 | 0.081 | -24.7 |
| RHO-PLGA/PEI | 157 ± 0.19 | 0.083 | +32.0 |
| RHO-PLGA/PEI/HA | 184.3 ± 2.6 | 0.100 | -22.7 |

A


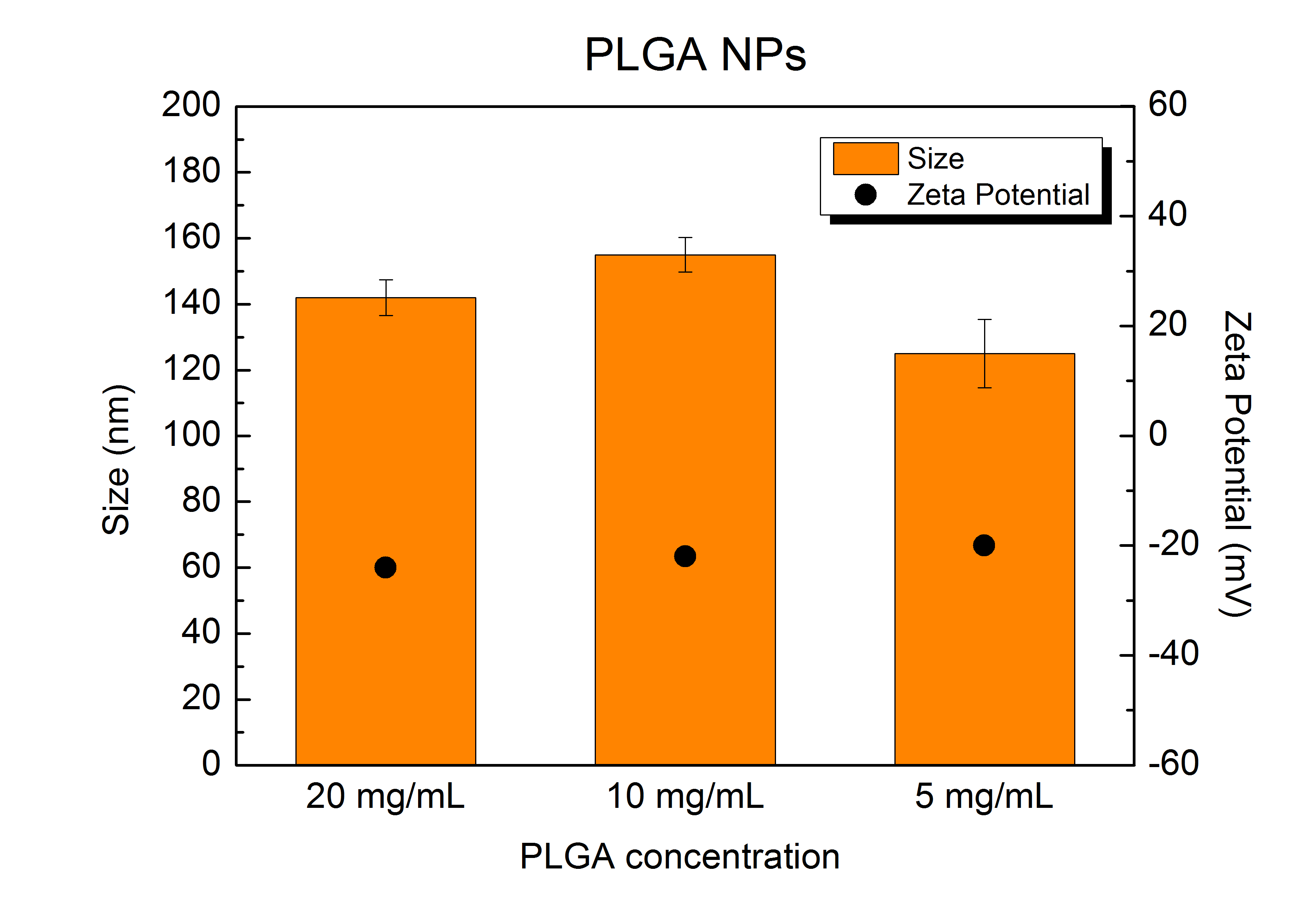


B

**
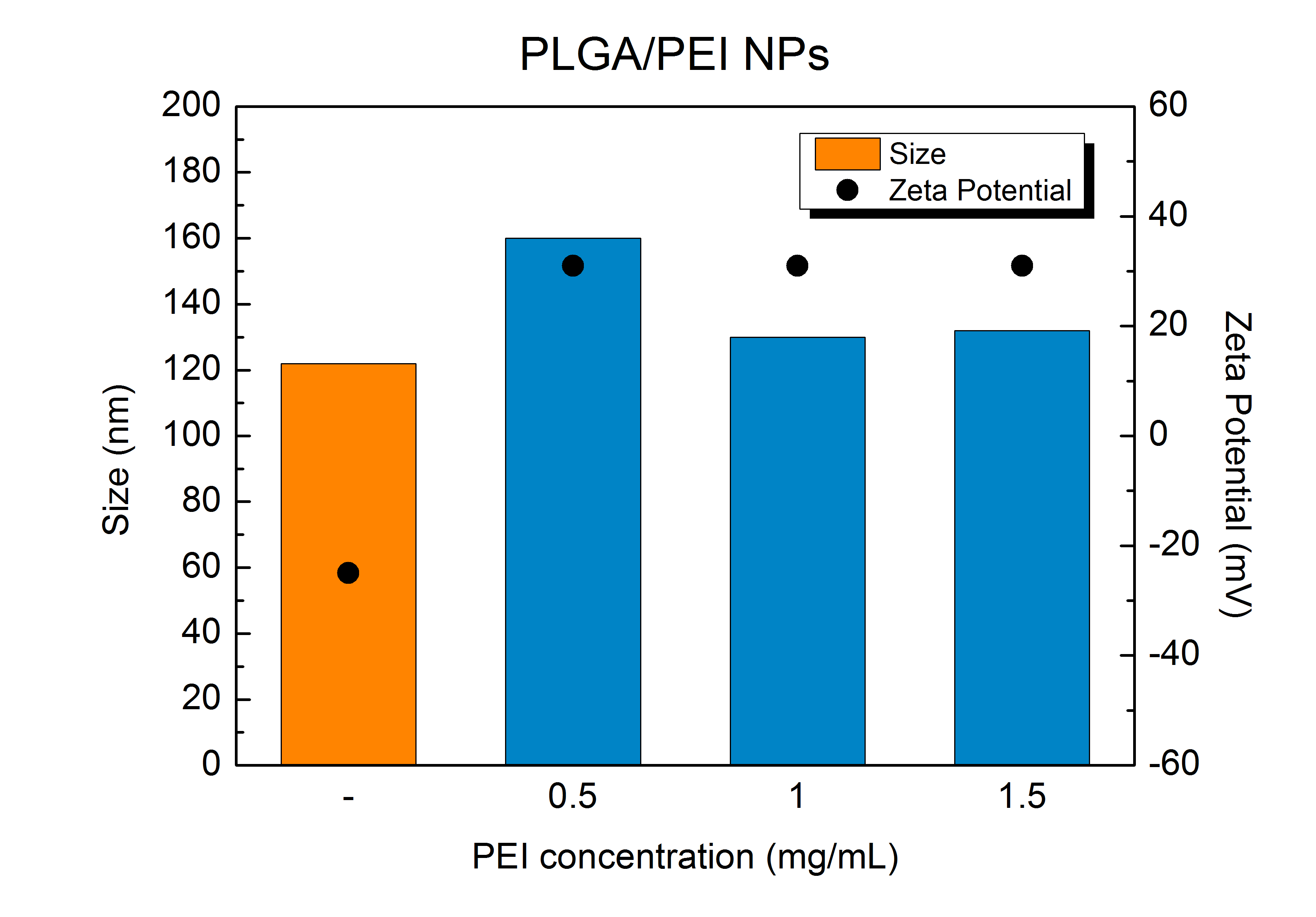
**

**Figure S1**. Effect of formulation conditions on the properties of PLGA core template. A) Effect of PLGA concentration. B) Effect of PEI concentration.

**
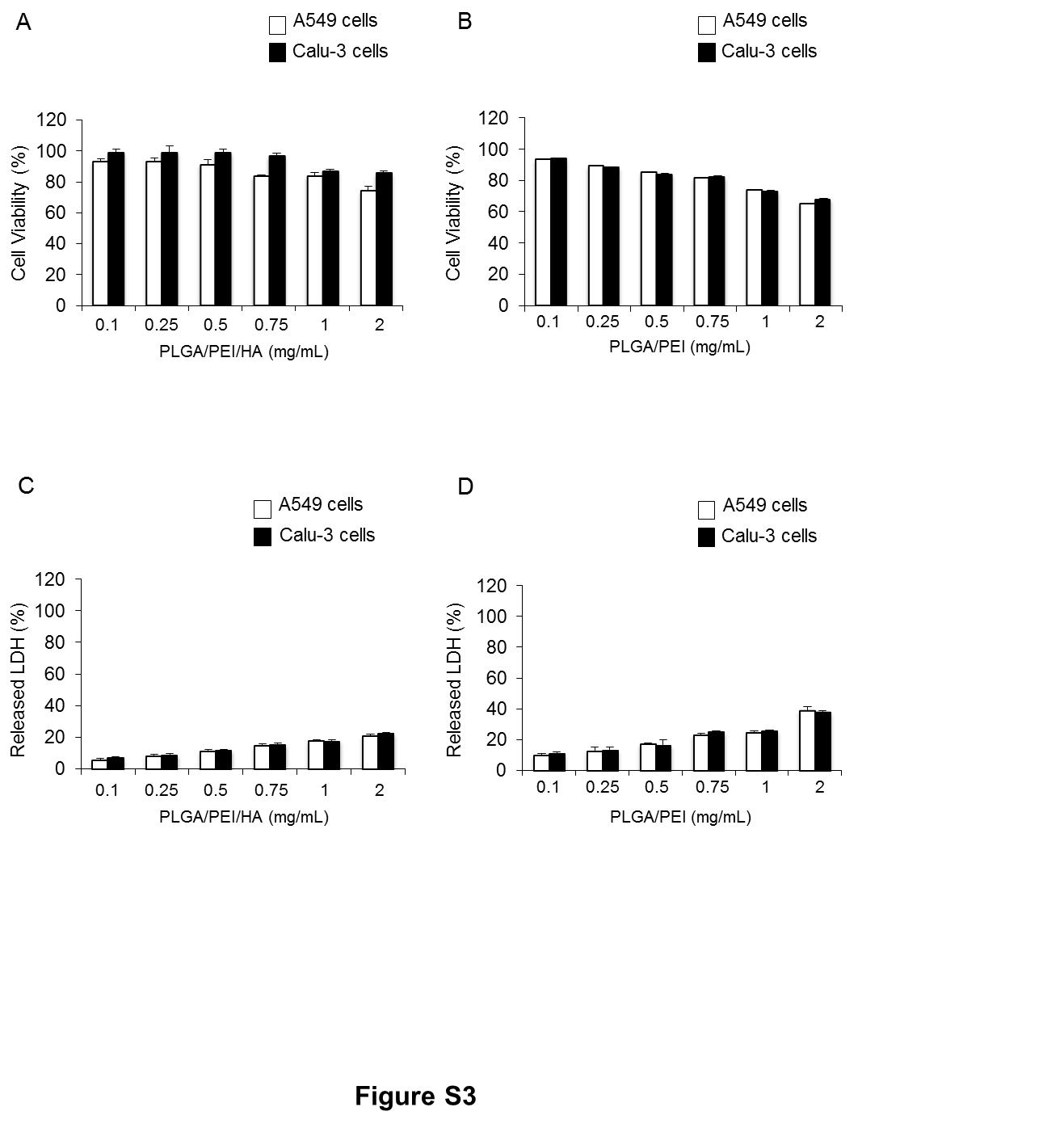
**

**Figure S2.** A549 and Calu-3 cells were exposed to increasing concentrations (0.1-2 mg/mL) of PLGA/PEI/HA NPs (A, C) and PLGA/PEI NPs (B, D) for 72 h. After incubation, cell viability and released LDH were evaluated using the MTT (A, B) and LDH (C, D) assays. The cell viability and LDH release from untreated control were set to 100% and 0%, respectively.

**
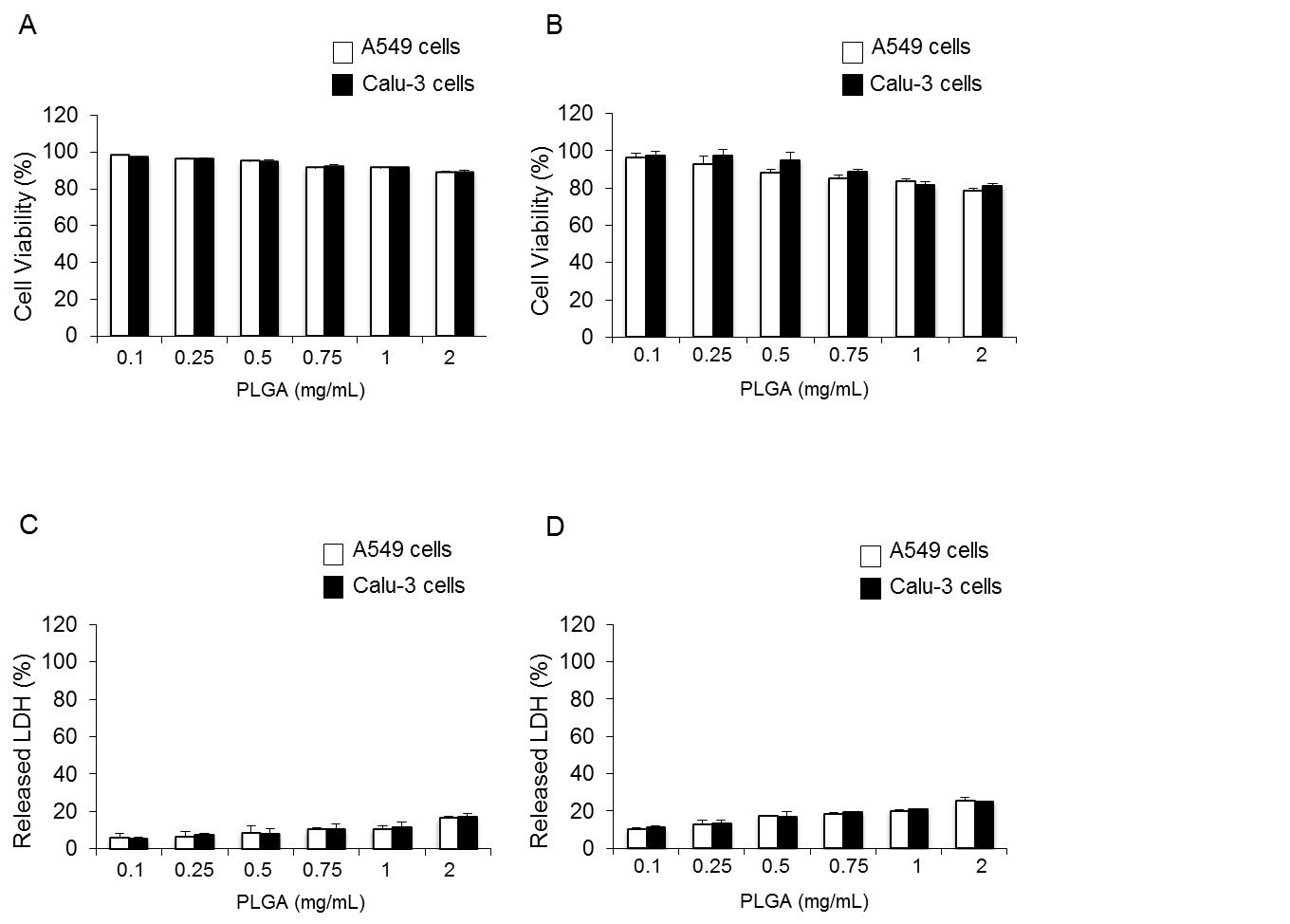
**

**Figure S3.** A549 and Calu-3 cells were exposed to increasing concentrations (0.1-2 mg/mL) of PLGA NPs for 24 h (A, C) and 72 (B, D) h. After incubation, cell viability and released LDH were evaluated using the MTT (A, B) and LDH (C, D) assay. The cell viability and LDH release from untreated control were set to 100% and 0%, respectively.

**Figure S4.** Structure of fluorescent PLGA-RHO polymer. Synthesis is reported in Maiolino S, Moret F, Conte C, Fraix A, Tirino P, Ungaro F, Sortino S, Reddi E, Quaglia F: Hyaluronan-decorated polymer nanoparticles targeting the CD44 receptor for the combined photo/chemo-therapy of cancer. Nanoscale 2015. [Epub ahead of print]. PMID: 25648974

**
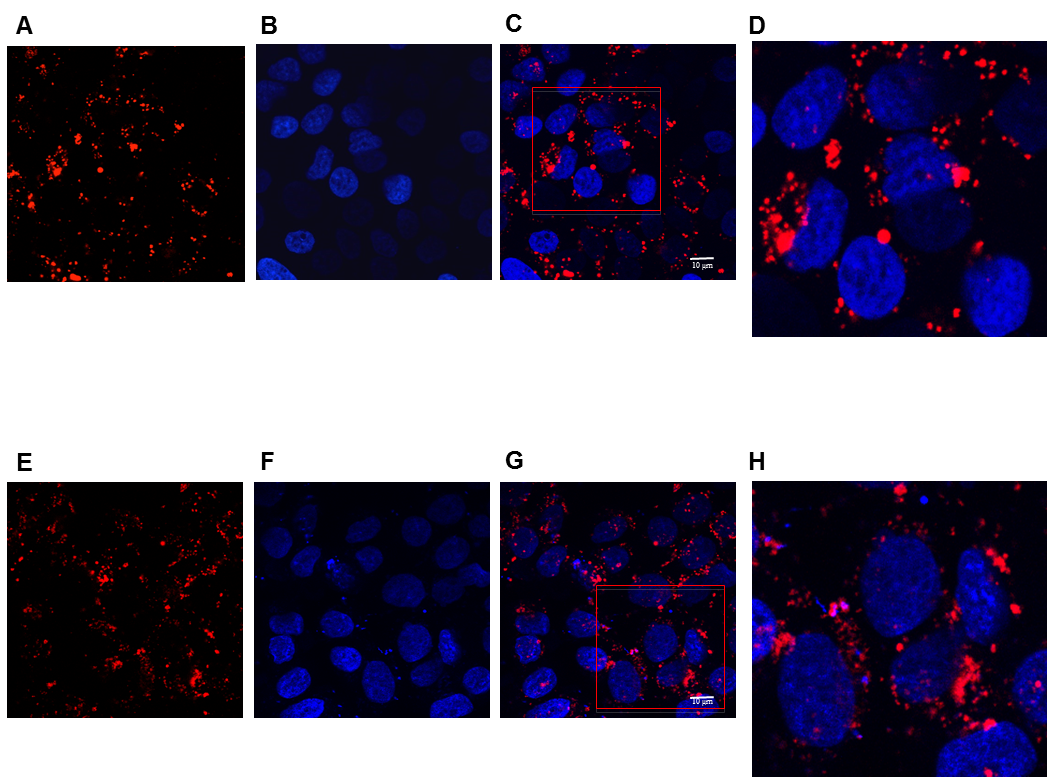
**

**Figure S5.** View of two fields of A549 cells incubated with RHO-PLGA/PEI/HA NPs (A) and RHO-PLGA/PEI NPs (E) for 24 h. Confocal microscope images 100X: A549 cell nuclei stained with DAPI (B, F). Merge of the same field for composite images (C, G), scale bar = 10 μm. Pictures were processed using ImageJ Software to reconstruct the x-axis projection using stack images (D, H).

**
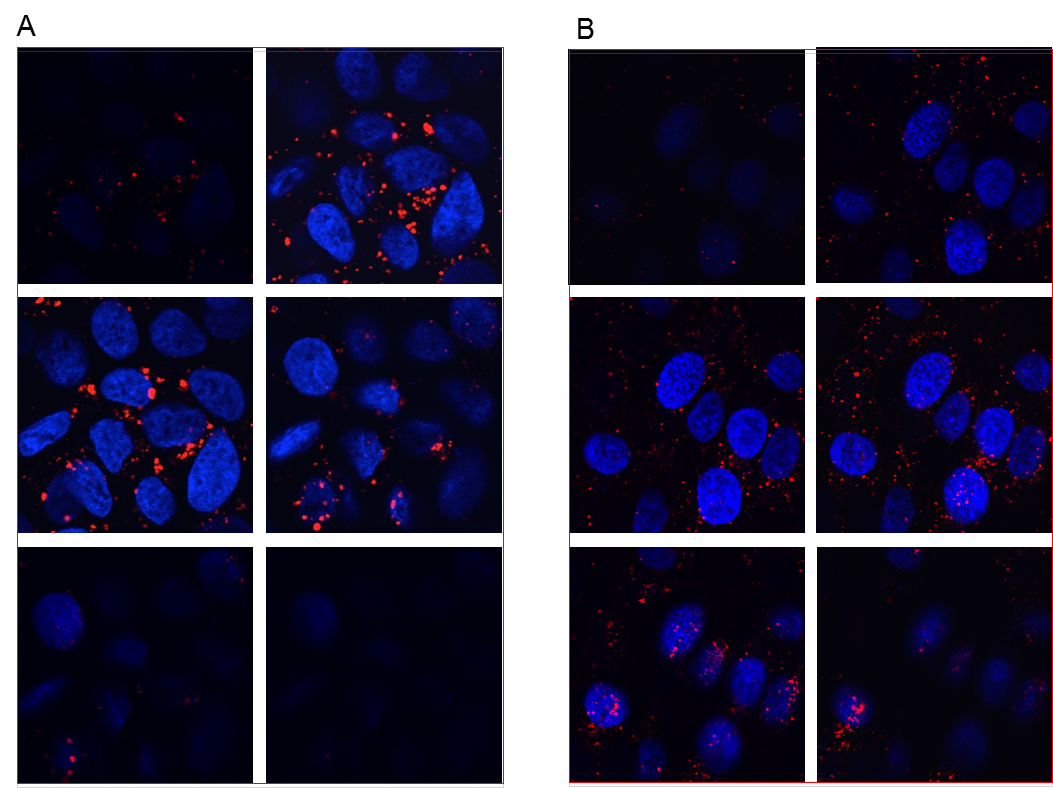
**

**Figure S6.** Analysis of z-sections taken through the cell nuclei of A549 cells incubated with RHO-PLGA/PEI/HA (A) or RHO-PLGA/PEI (B).

**
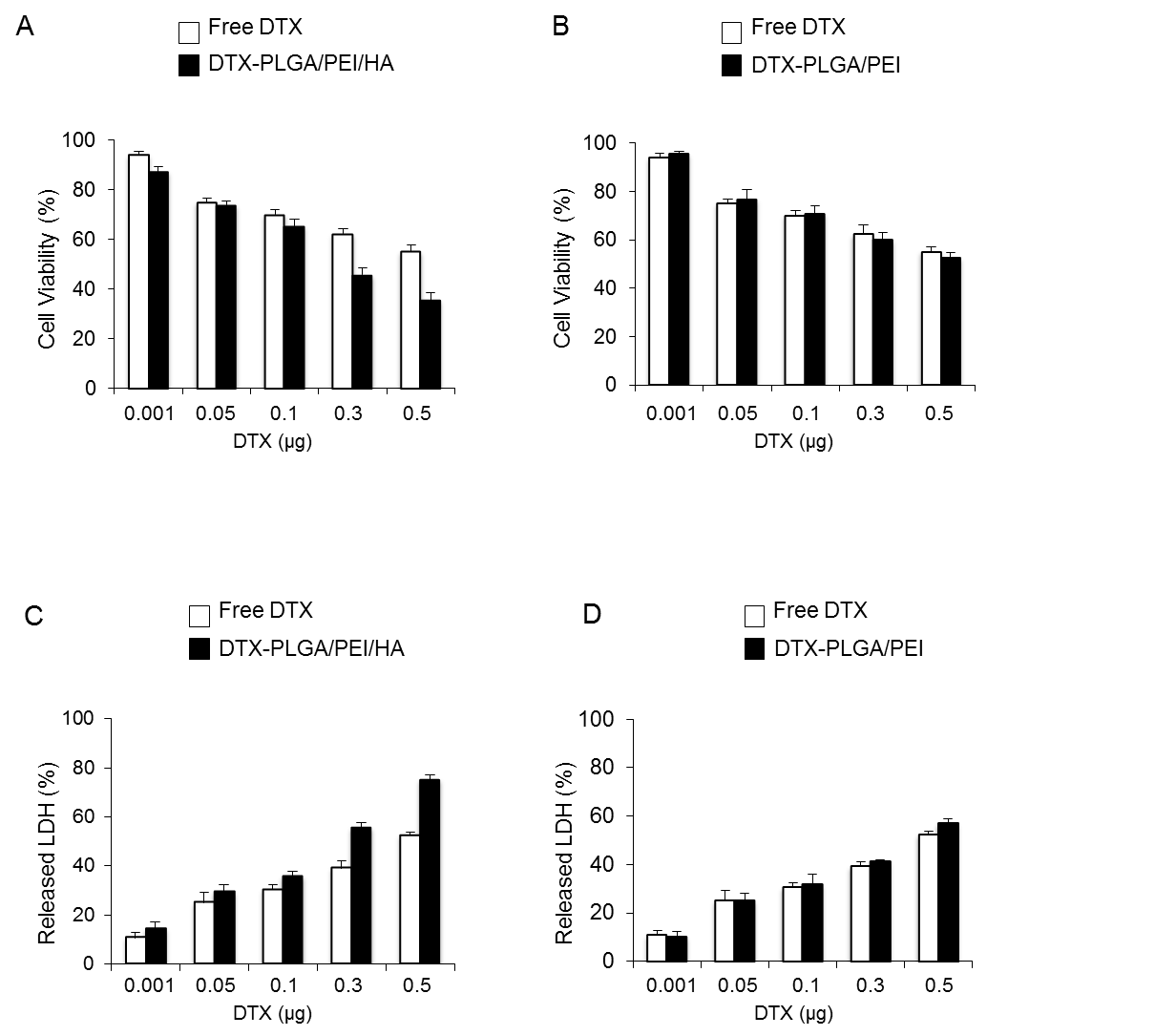
**

**Figure S7.** A549 cells were exposed to increasing concentrations (0.001-0.5 μg) of free DTX, DTX- PLGA/PEI/HA NPs (A, C) or DTX-PLGA-PEI NPs (B, D) for 24 h. After incubation, cell viability and released of LDH % were evaluated using the MTT (A, B) and LDH (C, D) assay. The cell viability and LDH release from untreated cells were set to 100% and 0%, respectively. Results are presented as percentage (mean ± SEM) (*n* = 3) of the control cells.
